# Process of care in outpatient Integrative healthcare facilities: a systematic review of clinical trials

**DOI:** 10.1186/s12913-015-0976-z

**Published:** 2015-08-12

**Authors:** Suzanne J. Grant, Jane Frawley, Alan Bensoussan

**Affiliations:** 1 National Institute of Complementary Medicine, University of Western Sydney, Locked Bag 1797, Penrith, NSW 2751 Australia; 2 Australian Research Centre in Complementary and Integrative Medicine, University of Technology Sydney, Sydney, NSW Australia

## Abstract

**Background:**

Patients currently integrate complementary medicine (CM) and allopathic, choosing a combination of therapies rather than a single therapy in isolation. Understanding integrative healthcare (IHC) extends beyond evaluation of specific therapies to encompass evaluations of multidisciplinary complex interventions. IHC is defined as a therapeutic strategy integrating conventional and complementary medical practices and practitioners in a shared care setting to administer an individualized treatment plan. We sought to review the outcomes of recent clinical trials, explore the design of the interventions and to discuss the methodological approaches and issues that arise when investigating a complex mix of interventions in order to guide future research.

**Method:**

Five databases were searched from inception to 30 March 2013. We included randomized and quasi-experimental clinical trials of IHC. Data elements covering process of care (initial assessment, treatment planning and review, means for integration) were extracted.

**Results:**

Six thousand two hundred fifty six papers were screened, 5772 were excluded and 484 full text articles retrieved. Five studies met the inclusion criteria. There are few experimental studies of IHC. Of the five studies conducted, four were in people with lower back pain. The positive findings of these studies indicate that it is feasible to conduct a rigorous clinical trial of an integrative intervention involving allopathic and CM treatment. Further, such interventions may improve patient outcomes.

**Conclusions:**

The trials in our review provide a small yet critical base from which to refine and develop larger studies. Future studies need to be adequately powered to address efficacy, safety and include data on cost effectiveness.

## Systematic review of clinical trials of integrative healthcare

*You think that because you understand* “*one” that you must therefore understand “two” because one and one make two. But you forget that you must also understand “and”.* —Sufi teaching story [1]

## Background

Patients are integrating complementary medicine (CM) and allopathic medicine in a variety of ways depending on their health status and health beliefs [2, 3]. They frequently use “bundles” of therapies rather than just one therapy in isolation [4, 5]. Typically the unit of integration in clinical environments is the patient rather than the practitioners [6, 7]. Increasingly consumers are requesting that there be improved communication and coordination between their allopathic care and CM providers [8]. In response to this consumer demand integrative healthcare (IHC), (also referred to as integrative medicine), has emerged over the past two decades [9, 10]. For healthcare policy makers, care providers, practitioners and the consumer, we need to know whether IHC is effective and, if so, what structures and processes combine to exert the positive outcome. Early experimental efforts have been made to investigate the effectiveness and efficacy of this evolving IHC model of patient care [11–13]. We sought to review the outcomes of recent clinical trials, explore the structure of the interventions and also to discuss the methodological approaches and issues that arise when investigating a complex mix of interventions in order to guide future research.

The varied definitions for IHC reflect differing interpretations and implementation [11, 14–16]. There is a common general underlying philosophy that IHC aims to treat the whole person (physical, emotional, energetic, spiritual), using the body’s innate ability to heal itself with a blend of conventional and complementary therapies [11, 14]. However, definitions of IHC splinter off when the structure and process of care are considered. For example, IHC may refer to the *process* of patient care where allopathic and CM clinicians work as a team. The *team* may operate in a multi-disciplinary or inter-disciplinary way. There may be a democratic referral system or the allopathic physician may be the gatekeeper and the CM practitioner is adjunct practitioner [17–19]. In other cases, ‘integrative’ may refer to a clinician who ‘integrates’ both allopathic and CM. Further confusion is added to the typology of this field by regional variations, where “integrated” is used in the United Kingdom (UK) and parts of Europe, in the same way in which “integrative” is used in the United States (US) and Australia.

The focus of this paper is IHC defined as a patient-centred, inter-disciplinary approach where there is a combination of conventional medicine with CM with shared patient assessment, treatment planning, review and/or shared practice guidelines that are constructed and utilised during the care process. This therapeutic strategy enables each practitioner, often in conjunction with the patient, to contribute their knowledge and expertise towards providing individualized healthcare plans.

In this emerging field, there are no ‘right’ or ‘wrong’ ways for patients to experience a combination of CM and allopathic medicine. Some conditions or people may be better suited to different processes of care. However, it is important to distinguish clearly between approaches which are adjunctive or complementary therapy and not integrative as they are different entities, have different organisational and resource implications, and likely, different benefits [14, 20]. Adjunctive or complementary is where a therapy is used in addition to allopathic medicine but not involving any shared assessment, management or review in the process of care.

Research methodology to evaluate IHC has tended to use observational designs, pre and post-test, or descriptive methods, but more recently there has been a move to the use of experimental design [21, 22]. IHC is a ‘complex intervention’, using experimental design with a complex intervention is challenging but feasible [23]. By ‘complex intervention’ we mean that there are many active components, which may combine to provide outcomes greater than a sum of its individual parts [11, 24]; that may involve complex mechanisms for delivery; may be difficult to replicate (tailored to individual); and may be influenced by the environment and social context. As such it is extremely challenging to clearly articulate mechanisms of action [23, 25]. IHC is often highly individualized, taking into consideration all aspects of a person not only one condition or symptom, has varied participant responses, with different practitioners in different settings [26, 27]. Determining the outcomes of a complex intervention requires a combination of qualitative and quantitative methods [23] and a consideration of the different experimental designs available [28]. In addition to measuring outcomes, understanding the complex intervention may be aided by including a nested process evaluation or similar. Process evaluations within trials explore the implementation, receipt, and setting of an intervention and help in the interpretation of the outcome results [24].

Hence, our review has three primary objectives:

1. To systematically review the quality and outcomes of clinical trials of IHC
2. To explore the design of IHC interventions, including process of care
3. To review research methodology employed in IHC clinical trials

## Methods

Eligible CM therapies were defined according to the National Institute of Complementary Medicine (NICM) [29]. NICM has adopted, with revisions, the four domains of CM articulated by the US National Centre for Complementary and Integrative Health (NCCIH). These domains are mind-body medicine, biologically-based practices, manipulative and body-based practices and energy medicine [30]. We included any clinical trial or protocol that was conducted in an IHC setting where there was a combination of at least one biomedical practitioner and one CM practitioner; and included an element of shared care or co-management of the patient at the stage of initial assessment, treatment plan development, case management or review. Clinical trials of multidisciplinary care where there was no element of co-management or patient sharing were excluded. Similarly, we excluded trials where the CM treatment was adjunctive and there was no shared management. Trials in integrative oncology were not examined in this review as the process of care in oncology settings differs from primary care settings, where the lead therapy is allopathic medicine and the lead clinician is the oncologist. Those conducted in an in-patient hospital setting were also excluded. Studies were also excluded which were not transferrable to a Western setting due to the different infrastructures in non-Western settings (e.g. Chinese medicine in China).

We searched PubMed (Medline), EMBASE, CINAHL, Clinical Trials Register and the Cochrane Library from inception to 30 March 2013. The following search terms were used, in various combinations: ‘integrated or integrative medicine’ or ‘healthcare’; ‘multidisciplinary care’; ‘complementary’; ‘alternative’; ‘biomedicine’; ‘conventional’; ‘mainstream’ with ‘medicine’; ‘healthcare’; ‘approach’; ‘therapies’. We also hand searched the citations of relevant papers. We included clinical trials which were randomized, non-randomized or case–control. For the purpose of this study we also included protocols for clinical trials, as we were not only focused on outcomes but on the structure and design of the integrative therapeutic intervention. Our search terms were [(Integrat* OR interdisciplinary) and (medicine OR alternat* medicine OR alternat* therap* OR complementary medicine OR healthcare)]; OR [(allopathic OR conventional OR mainstream OR orthodox OR biomedicine) AND (alternat* medicine OR alternat* therap* OR complementary medicine OR intergrat*].

To explore the structure and design of the IHC intervention we applied the structure-process-outcome model [31]. Structure was defined as the environment in which healthcare is provided, the process as the method by which healthcare is delivered and outcome as the consequence of the healthcare provided. We were particularly interested in the process component of this model. We defined process as the way in which the healthcare is delivered – triage, diagnosis, treatment plan, and review. Central to this process are the means for collaboration to foster the “integrative” nature of the intervention. These concepts are explained below:

1. ‘Triage’ in this context refers to how a patient is ‘allocated’ into an IHC intervention or in a clinical context to a practitioner for initial assessment. In clinical practice the gatekeeper for this process may be the receptionist, the practice nurse, or general practitioner (GP) or may come from an external referral.
2. ‘Diagnosis’ refers to initial assessment where baseline data is collected in a clinical trial or where an IHC assessment is undertaken in clinical practice.
3. ‘Treatment plan’ refers to how the plan is derived, who is consulted, how it is agreed, types of treatments, duration, patient preferences and arriving at responsibility for the patient’s journey through the IHC process.
4. ‘Review’ refers to measuring outcomes at set time points, tracking patient compliance,
5. ‘Means for collaboration’: this may include meetings, shared charting, electronic medical records (EMR), corridor conversation, shared education and training, case conferences.

To review the research methodology, we considered the Medical Research Council (MRC) guidelines [23] for evaluating complex interventions and extracted data on the following:

- Was there a theoretical basis for the intervention and trial design?
- ave the authors sufficiently described their intervention and the control group?
- How was the intervention evaluated [32]?
- What were the context and the environment of the intervention?
- How was implementation of the intervention assessed?
- What was the rationale for the duration of the intervention?

Two investigators (SG and JF) independently screened all identified titles and abstracts. Full text reports were retrieved. The final decision for inclusion in the review was made by two investigators (SG and JF). The quality of the trials was also reviewed using risk of bias criteria [33]. Each of the retrieved papers was read in its entirety. A standardized data extraction form was developed to extract data according to the set elements. Where data was missing we attempted to contact authors of the study. Data elements were then extracted and entered into a matrix according to the method of Garrard [34]. This approach provided a systematic and concise organisation of the literature.

## Results

**Fig. 1:**
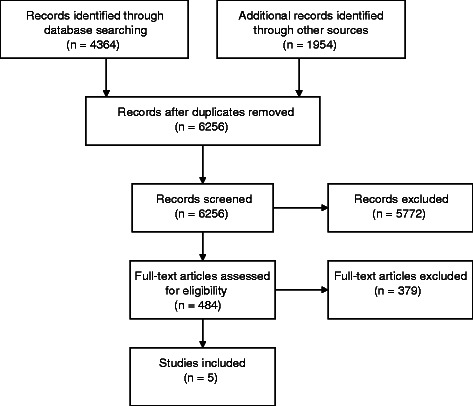
PRISMA flow chart of study selection

A total of 6256 papers were screened. Of these 5772 were excluded based on the title and/or abstract. The main reasons for exclusion were not relevant (*n* = 4865), study of CM usage or attitudes to CM (*n* = 304), and conceptual paper or commentary (*n* = 258). A total of 484 full text articles were retrieved for consideration. A Preferred Reporting Items for Systematic Reviews and Meta-Analyses (PRISMA) flow diagram (Fig. 1) shows details of the study selection. Papers were excluded where the intervention or clinical practice was a set multi-disciplinary program or CM was adjunctive not integrative and there was no shared management (*n* = 49), the article was about CM usage (*n* = 296), the setting was oncology or inpatient hospital based (*n* = 149), the paper was conceptual or commentary (*n* = 117), examples of IHC clinical practice (*n* = 63), or limited to a discussion of models of IHC (*n* = 28). Five clinical trials met the inclusion criteria. We excluded the six clinical trials that had been included in the previous review [19] as they did not meet our criteria of integrative management or were hospital-based programs.

### Study design and setting

Four randomized controlled trials (RCT) and one case control study met the inclusion criteria of our study. Table 1 outlines the study settings and trial designs. Three studies were conducted in the US, one in the UK [35] and one in Sweden [36]. There was one three arm trial comparing, IHC with chiropractic and medical care, and with conventional medical care alone [37]. Two were pragmatic trials comparing individualised, integrative care with usual care [36, 38, 39]. The study in the UK was quasi-experimental design with a wait-list control group [35].

**Table 1:** Characteristics of IHC Controlled Clinical Trials

Author, Ref	Sample, Diagnosis & Setting (Trial design)	IHC Intervention (Duration)/ Comparator	Outcomes measured	Results
Westrom et al. 2010; Maiers et al. 2010; Bronfort et al. 2012; Westrom et al. 2010 [39, 41, 44, 89]	201 adults with LBP ≥ 6 weeks (RCT).	Integrative, multidisciplinary care: acupuncture, oriental medicine, cognitive behavior therapy, exercise, massage, chiropractic and/or medicine (12 weeks)	Patient Self-Assessment Form (PSAF is a modified form of MYMOP); Frequency of symptoms; RMQD modified; Fear avoidance beliefs questionnaire; Pain Self Efficacy Questionnaire; EuroQol 5D, improvement pm a 9 point ordinal scale, satisfaction, work loss, and medication use; also Lumbar dynamic motion and Torso muscle endurance. Semi-structured interviews with both patients and providers at the end of the study.	IHC group statistically significant improvement in pain reduction, perceived global improvement and satisfaction with care.
		Vs		
	*Setting*: Wolfe Harris Centre for Clinical Studies, Northwestern Health Sciences University, Minnesota, USA	Chiropractic care alone.	*Assessed:* wks 12, 52	
Eisenberg et al. 2012 [38]	20 adults with LBP for 3–12 weeks in the US (RCT, Pilot)	Integrative, individualized care: acupuncture, chiropractic, massage, occupational therapy, physical therapy, mind-body techniques, neurology consultation, nutritional counselling, orthopaedics consultation, and psychiatry and rheumatology consultation and referrals as appropriate, plus usual care (12 weeks). Treatment provided up to two times per week, with up to two treatment modalities per session.	RMQD; Symptom relief using Bothersome index past 24 h; Pain past 24 h; SF12; Worry	Week 12 Roland Morris (*p* < 0.08); bothersome (*p* < 0.02); pain (0.005)
	*Setting*: multi-speciality group practice, and an academic teaching hospital (Outpatients), Boston, USA	PLUS Usual Care	*Assessed:* wks 2, 5, 12, 26.	Preliminary findings: significant difference in favour of the IHC group on pain reduction, perceived global improvement at 12 and 26 weeks.
		Vs		
		Usual care alone including medications, referral for physical therapy as needed, education, limited bed rest and activity alterations		
Goertz et al. 2013 [37]	120 sub-acute or chronic LBP of at least 4 weeks duration in adults ≥ 65 years (RCT, Pilot).	Collaborative medical, osteopathic and chiropractic care who comprise a patient-centred, co-management team (up to 12 weeks)	Primary outcome self-report LBP on a 11-point numerical rating scale (NRS); RMQD; SF36 (Veterans RAND); FABQ; Functional mobility with Timed Up and Go test; symptom bothersomeness index past week; depression and anxiety (Patient Health Questionnaire-9 for depression; General Anxiety Disorder −7); Self-report healthcare utilisation, expenditure and medication use; Questionnaire to assess expectations, improvement, satisfaction; Specific process outcomes: participant and provider perceptions of the collaborative care model and the clinical trial design.	Protocol only, trial underway. This is a pilot to assess and refine the collaborative care model and the sample size has not been calculated to detect a significant difference on the outcome measures.
	*Setting:* Chiropractic research clinic and Family Medical Centre, USA	VS		
		concurrent medical and chiropractic care provided by an unlinked family medicine physician and a doctor of chiropractic	*Assessed:* Baseline, 4, 8 and 12 weeks (primary endpoint), and every 12 weeks after up to one year.	
		VS		
		conventional medical care provided by a family medicine physician		
Sundberg et al. 2009; Sundberg et al. 2007 [36, 46]	80 adults with back/neck pain of at least 2 weeks duration (RCT).	IHC involving an individualized treatment plan provided by a multidisciplinary IM team coordinated by a gate keeping GP. Therapies included seven sessions of a selection of the following: massage, manipulative therapy, shiatsu, acupuncture, qigong (group based) for a period over 10 weeks.	SF36; IM tailored outcomes targeting self-rated disability, stress and well-being; Days in pain (0–14); Healthcare utilisation and medication use. Focus group discussions exploring participants’ experiences and perceptions of conventional and integrative care.	Significant improvement in one (vitality) of the eight domains of the SF-36. Trend to less medication use in the IHC group. Underpowered.
	*Setting:* IM clinic operating 5 days per week at a primary care unit in Sweden 2003-2006	PLUS Usual Care	*Assessed:* Baseline, 12 weeks, 16 weeks (by post)	
		VS		
		Usual care		
Richardson et al. 2001a; Richardson et al. 2001b [35, 40]	330 adults with over 20 conditions (quasi-experimental).	Integrative, multidisciplinary, individualized care: Acupuncture, Homeopathy, and Osteopathy for six treatment sessions up to 12 weeks.	SF36 baseline and at completion of treatment. Patients were asked about their satisfaction and experience of the service.	Significant improvements in the intervention group in seven of the SF36 eight domains.
		VS		
	*Setting:* Complementary Therapy Centre set up within a hospital, UK	Waitlist		

*RMQD* Roland Morris Disability Questionnaire, *SF12* and *SF36* the Short Form (12 or 36) Health Survey, *FABQ* Fear Avoidance Beliefs Questionnaire

Settings varied; three were family or primary care medical centres and the others were conducted within an academic setting (Northwestern Health Sciences University) or out-patient hospital setting. In the Sundberg [36] study, an IHC clinic was set up within a primary care unit in Sweden. Patients were treated within this clinic or in some cases treatments were provided off-site in the private clinics of participating providers. Participants were required to pay five Euros per treatment for the first six treatments, after which no further payment for CM would be required. In the Richardson study [35], patients were treated within the context of a CM outpatient service operating in line with other National Health Service (NHS) outpatient provisions in the UK. In all other studies treatments were free of charge.

### Participants

Four of five studies were conducted in people with lower back pain (LBP), with one study treating neck pain in addition to LBP [36]. The fifth study was a case control study that investigated the treatment of referred patients with over 20 different conditions, although the majority of the patients treated presented with neck/back pain [35]. All studies were conducted in adults and enrolled participants with diverse backgrounds and co-morbidities. A total of 751 people were involved in the studies and sample sizes ranged from 20 participants in a pilot study to 330 adults in the case control study.

### Process of care: Triage and diagnosis

Four of the five studies published at least one preliminary paper outlining the background of the study [40], the protocol [7, 37], the clinical care pathway [41] or the model [42] to be used in the clinical trial. In Table 2, the initial assessment process is outlined. In two studies, the initial assessment was conducted by a single allopathic physician [36, 40]. In the other three studies, one study used an allopathic physician and a CM practitioner together [38]; another used assessments conducted by a combination of either a chiropractor, an osteopath or a medical doctor [37]. The initial assessment was reported in detail in two of the studies [37, 39].

**Table 2:** Process of care in IHC trials

Author, Ref	Initial assessment	Treatment Plan	Means for Integration and Collaboration	Cost effectiveness
Maiers, Westom, Bronfort et al. [39, 41, 44, 89]	Patient completes a baseline evaluation profile comprising self-report back pain symptoms, disability, general health status, fear avoidance, self-efficacy measures and patient perspectives (previous experiences with LBP treatments, preferences for care and expectations of study treatment) as well as physical exam and objective test findings.	One or more treatment plans are developed for the patient at a weekly meeting. Each treatment plan consists of one or more modality and consensus must be reached for the plan to be presented to the patient. Typically there are three care plan options consisting of two to three modalities.	Clinicians attended one full day training prior to commencing study. Training included:	Cost effectiveness analysis between intervention groups using ICER and a cost utility analysis based on the EuroQoL5D from a societal perspective.
	Before randomisation, the profile is reviewed by multidisciplinary team during weekly case review meetings to determine eligibility. A second baseline evaluation visit where patients are enrolled and complete baseline measures. Once randomized, patients are discussed at weekly meetings.	Care consultation with patient conducted by non-clinician case managers where treatment plans were presented and patient exerts preference for a plan.	information on each healthcare discipline;	
		At weekly meetings, clinicians review patient progress using the PSAF, self-rated symptoms and activity against benchmarks of expected improvement. If progress not satisfactory, a patient’s profile may return to team meeting for consideration of changing the treatment plan. Facilitated by specifically trained non-clinician case managers.	review of the available clinical evidence on the effectiveness of each modality when used to treat LBP;	
			applying an evidence informed practice model;	
			methods for reaching consensus in a team.	
			Ongoing training as needed throughout the study.	
			Site visits by consultant to observe team dynamics and provide feedback.	
			Weekly meetings facilitated by non-clinician.	
			Shared access to treatment notes.	
Eisenberg et al. [38]	Allopathic doctor and CM clinician evaluate the patient together.	The two evaluators meet to develop an individualized treatment plan. Treatment plan initiated by appropriate clinicians.	Team trained one full day per wk for 14 weeks prior to study. Co-led by a professional facilitator, a medical anthropologist included:	Maximum outcomes with minimum treatment. Number and frequency of visits recorded but no cost effectiveness component included in the study.
		At team meetings, cases are presented and discussed for input from all members and treatment plan modified by team’s recommendations. This process was ongoing.	Presentations by each member	
			Experiential education including hands on diagnosis and treatment by each member on other team members	
			Diagnosis and treatment of volunteer subjects with chronic LBP	
			The development of a shared treatment protocol for the implementation of the pilot RCT.	
Sundberg, Andersson et al. [36, 46]	Allopathic doctor served as gatekeeper with responsibility for overall management of the patient – only licensed medical doctors are permitted to fully utilise the complete range of medical services. The allopathic doctor had clinical knowledge and experience of CM.	Consensus case conference with CM providers to identify appropriate treatment strategies tailored to the patients’ needs.	Regular team meetings in the lead up to and during the project Training to work collaboratively, utilise a consensus case conference model within primary care, meeting included:	Patients charged a low fee per treatment and low maximum treatment cost to obtain all treatments. No adverse events. The IM model, on average integrating 7 CT sessions with conventional primary care over 10 weeks, resulted in increased QALYs, somewhat higher cost of healthcare provision but a reduced cost of using healthcare resources, including less use of analgesics compared to conventional primary care. The costs/QALY ranged between euro 24 000 and 41 00There was a conservative likelihood of the IC model being cost-effective at a threshold of EUR 50,000 per quality-adjusted life year gained.
		Initial conference followed by regular consensus case conferences combining conventional and CM clinical reasoning to verify and improve clinical management of patient.	Professional presentations	
		Aimed for non-hierarchical decision making.	Educational items on different medical models	
		Patients did not participate in the consensus case conference but via personal interaction with IM provider.	Case management strategies (approaches to diagnosis, treatment, prevention and documentation)	
			Used a medical record developed specifically for the trial	
Goertz et al. [37]	Doctor of Chiropractic gathers history, conducts eligibility examination including mobility assessment, fracture risk, reviews scores for depression, anxiety and substance abuse, and requests any additional information such as x-rays. All data recorded on web based form and reviewed by other physicians and patient attends second eligibility exam with Doctor of Osteopathy or medical doctor. Case review sessions held twice weekly with DoC and study coordinators present to agree on inclusion. Patient is then randomized.	Team of clinicians assigned to case to follow during 12 weeks.	To prepare for “Shared Care” clinicians completed a 6-month interprofessional educational program comprised of advanced training in LBP both medical and chiropractic, imaging studies and LBP in older adults. Interdisciplinary discussions on simple and complex cases for LBP suitable for co-management	No.
		Interprofessional telephone consultation to discuss patient and establish treatment plan.	To foster interdisciplinary practice during the study:	
		Treatment plan communicated to patient by next treating practitioner	research record sharing via a secure electronic Doctor Communications module specifically constructed and maintained for the study within a web-based tracking and reporting system;	
		Team based case management:	interprofessional telephone consultations;	
		Additional telephone call consultations or research record exchanges may be initiated to change treatment plan, refer as warranted	patient centred treatment planning and evaluation	
			half day site visit at partner clinic to shadow one or more practitioners involved in trial	
			quarterly interprofessional education sessions	
Richardson et al. [40]	A pilot service run by a consultant physician and managed by a service manager, coordinated on a daily basis by a senior staff nurse who was also qualified in massage. Patients referred to the service by local GP and hospitals. The GPs act as gatekeeper and refers to the service. Referral guidelines were developed through consensus conference of 27 health professionals. The referral table lists over 20 conditions suited to one of the three therapies available and contraindications. GPs were the gatekeeper for referral to treatment, and used the referral table for guidance. Patient preference unclear.	Unclear. Staff meetings regularly held and audits conducted but not clear if these discussions altered the patient treatment plan.	The initial Delphi process involved a half day discussion of conditions, therapies and the indications of each for 26 health professionals.	No
			Shared bespoke computer system for patient demographic and clinical information.	
			Practitioners discussed cases in staff meetings which were attended by the medical director, clinic nurse and other practitioners. Local GPs were involved in case presentations where possible.	

*PSAF* Patient self-assessment form

In the Maiers’ [41] study, an initial baseline profile was compiled by research study staff. This evaluation included informed consent, self-report questionnaire, health history, physical examination and x-rays if indicated. The self-report questionnaire includes patient perspectives such as previous experience with treatment, preferences and expectations. This profile was reviewed by the IHC team and a second visit scheduled and then assessed by the multidisciplinary team for eligibility [39] If eligible the patient then completed a second baseline visit, was enrolled and randomized to a treatment group.

The Goertz [37] study similarly used research study staff to conduct an initial baseline evaluation to gather back pain and chiropractic treatment history, beliefs about chiropractic, health assessments and a treatment expectations questionnaire. Once eligibility of the patient was confirmed by all physicians, a second baseline visit conducted by a medical or osteopathic physician with a focus on age-related concerns and the participant is then randomised to conventional care, dual care or shared care.

In the Sundberg [36] study, after an initial consultation with a GP at the primary care unit, a conventional treatment plan was developed for every participant. The research team randomised participants to receive either integrative care or continue with conventional care only.

In the Richardson [35] quasi-experimental study, referrals were accepted to the study from general practitioners (GPs) and hospital doctors and patients. In the Eisenberg [38] study, participants were initially assessed by both a medical doctor and a CAM clinician.

### Process of care: treatment plan

Treatment plan refers to how the plan is derived, who is consulted, how it is agreed, types of treatments, duration, patient preferences and arriving at responsibility for the patient’s journey through the IHC process. Table 2 provides some detail on how the treatment plans were derived. Four of the studies used a team approach to developing one or more treatment plans for each patient [36–39]. In the team approach, typically non-hierarchical, consensus based decision making was sought. None of the case conferences or team meetings to develop treatment plans involved patient participation.

Patient preferences were considered in two studies. In one of these studies, the outcome of the team approach was typically three treatment plan options that were then presented to the patient by a non-clinician case manager. The patient was then able to express their treatment plan preferences [39]. In the other study, a treatment plan was communicated to the participant and participant feedback was incorporated into the management plan [37].

Oversight for the patient’s journey in the trial was not always clearly documented. In one study it was clearly the general practitioner (GP) [36], as only medical doctors in Sweden are able to access the full range of medical services. In the UK study, a nurse coordinated the patient’s journey through the study [35].

The type of therapies and combinations were comprehensively documented in two of the three fully reported studies [41, 42]. In the Maier’s study, manual therapy, exercise and education were the most commonly recommended and selected modality combination followed by a combination of exercise therapy, education and oriental medicine. An average of 3–4 (3.4) treatment plans were offered to each of the 101 participants, with an average of three modalities per plan. Sundberg et al. documented that patients received around seven treatments, averaging two different modalities over 10 weeks along with one session of a self-help activity – qigong. Swedish massage was the most commonly combined modality.

All study interventions involved one to two treatment sessions per week with administration of up to two treatment modalities per session. The interventions included combinations of different practitioners and therapies that were deemed appropriate to the condition being treated. The four studies on LBP typically utilized differing combinations of acupuncture, osteopathy, chiropractic, massage, mind body techniques and/or exercise. The case control study used acupuncture, homeopathy and osteopathy as therapies.

The study comparator was usual care in three studies [36–38]. The case control study used a waitlist for comparison, whilst another study compared the IHC intervention to chiropractic treatment alone [39].

The length of the intervention was 10–12 weeks for all five studies and none of the studies documented any data collection of co-morbidities or treatments for co-morbidities. Only one study provided a rationale for the length of the intervention, hypothesizing that a 12 week intervention period was typical in treating the study population [41]. One study had a follow up at 16 weeks [42] and another study had a follow-up period of 52 weeks [37].

### Process of care: Review

All studies had a review process built into the intervention, in some cases a formal process was in place for review and in others cases were only discussed at weekly staff meetings and informally between practitioners [40]. Re-evaluations were common and of the 101 treatment plans, 38 were re-evaluated during the course of the study. The main reasons for re-evaluation were due to lack of improvement as perceived by the provider, patient or both. Maiers et al. used a modified Measure Yourself Medical Outcome Profile (MYMOP) to assess and modify treatment during the intervention period. This study also used benchmarks for improvement in the condition and the case was returned to the team if benchmarks weren’t met [41].

### Process of care: Means for integration and collaboration

Educational sessions or training of therapists and practitioners was conducted in all of the studies prior to commencement [35, 37, 38, 41, 42]. This ranged from a half day workshop [40] to a six month course in inter-professional education [37]. In another study, the team met weekly for one full day for 14 weeks to undergo training [38].

Training typically comprised knowledge sharing of the different modalities to be used in the study, often involving presentations by each of the practitioners and in some cases hands on experiential sessions. Evidence for modalities was also reviewed in these sessions. Four studies referred to sharing patient medical records and treatment notes. In some cases specific computer modules or systems had been constructed specifically for the study.

Team meetings, either face-to-face or via phone, were also seen as a regular part of the integrative approach. It was implied in four of the studies that the treatment plan may be altered as a result of these meetings but this process was only clearly detailed in two of the studies [38, 39]. In one study, patient progress was reviewed using outcome measures being assessed against benchmarks and if progress was not satisfactory the treatment plan was modifies.

Four of the studies [36, 37, 39, 40] referred to using an existing evidence base to guide treatment. Evidence for effectiveness was typically tempered with clinical experience and patient presentation. In some cases, the evidence directed the modalities that were selected for inclusion [37, 39] or guided the referral process [40]. A care pathway was designed for one study. This provided a structured process for clinicians to follow including points along the way to flag treatment review.

### Outcome measures and results

Only one of the studies was powered to seek efficacy outcomes [39] and three trials were pilot studies [36–38]. The objective of the case control study was to establish the feasibility of an IHC service as opposed to determining the efficacy of the service.

The results of the adequately powered study showed that IHC in people with LBP resulted in statistically significant improvements in pain reduction, perceived global improvement and satisfaction with care compared to those patients receiving chiropractic care alone [43, 44]. Quality of life was rated as significantly better in the IHC group compared to the chiropractic group at 52 weeks. The feasibility and pilot studies all reported favourable trends towards the IHC group.

Condition specific outcome measures such as a pain scale or validated disability questionnaire such as the Roland Morris Disability Questionnaire (RMQD) were used in four of the five studies [36–39].

All but one study also used a standardized quality of life instrument. Only one study [41] used a patient generated measure, a modified MYMOP [45]. Generalizable cellular or molecular markers such as inflammation markers were not collected in any study.

Practitioner and/or patient expectations and perceptions of the integrative care approach were reviewed through semi-structured interviews in four of the studies [35, 37, 39, 42]. Patients found the holistic approach of integrative care to be empowering, facilitating self-help in contrast to the conventional managements which was disease focused and reductionist, but were concerned about the challenges of treatment costs [46]. Two other studies are yet to fully publish their qualitative results. The Goertz [37] study has conducted semi-structured interview to ascertain participant’s perceptions of back pain improvement, clinical trial experience, expectations, treatment effects, and interprofessional collaboration amongst the treating clinicians. Clinician expectations of care were also recorded along with their thoughts on expected improvement of overall quality of life and health status and their rationale for the treatment provided.

### Cost effectiveness

Crude cost data was collected in one study only [36]. Extra costs of treatment were calculated at 365€. In this study there was a trend to decreased prescription and non-prescription medications. The cost of the treatment only was collected in one other study but has not been reported [39].

### Safety

One minor adverse event was reported out of 170 IHC interventions provided in one study [38].

### Quality of trials

Four of the five studies were randomized controlled trials. Table 3 provides a brief assessment of the risk of bias of the studies. All trials used appropriate methods of randomization (computer generated) and reported allocation concealment. Blinding of care provider and participants is not possible in an IHC intervention, although in some cases it is possible to blind the outcome assessment. Only one trial reported blinding of outcome assessors where questionnaires were administered by research staff not involved in clinical care. The risk of bias of the RCTs is low if the difficulty of blinding is considered. Blinding of participants and care providers in a trial where treatment is individualised is not possible. However, outcome assessment may have been blinded, one study achieved partial blinding of outcome assessment [46].

**Table 3:** Methodological quality: Risk of bias

	Westrom	Goetz	Eisenberg	Sundberg
Random sequence generation	Low	Low	Low	Low
Concealment of allocation	Low	Low	Low	Low
Blinding of care provider	High	High	High	High
Blinding of participants	High	High	High	High
Blinding of outcome assessment	High	High	High	Partial
Incomplete outcome data	n/a	n/a	Low	Low
Selective reporting	n/a	n/a	Low	Low

The case controlled study had no randomization [35]. Patients were treated on a first come, first serve basis and urgent cases were given priority. To address the bias inherent in such a design the authors followed a quasi-experimental approach for non-equivalent groups without controlled selection. Sensitivity analysis was conducted on the urgent referrals, and the poor response in the waitlist group. This waitlist control study has limitations due to expectation bias, and selection bias. There are also limitations due to the complexity of the intervention and evaluation of the effect applies to the whole treatment and not to the individual components.

### Review of the research methodology

All studies provided an adequate rationale for the intervention and trial design [36–40]. The Richardson [40] quasi-experimental study was based on a pragmatic approach and used the convenience of a ready-made waiting list. The rationale for all of the studies was primarily 2-fold: patients are currently integrating various CM; and the value of this integration remains largely unknown in terms of outcomes and cost effectiveness.

Table 1 provides a summary of the interventions and comparators used in the studies. The therapies available and the comparators are adequately described but the dosage is not always apparent. In only two of the studies have the authors sufficiently described the number of modalities and frequency of sessions of the intervention on average for the participants [36, 41].

Outcome measures detailed in Table 1 show all studies sought to evaluate the interventions using a quantitative measure for quality of life such as SF36 and a symptom specific tool. All but one study [38] used interviews to explore participant perceptions of the IHC model and two studies also included interviews with providers [37, 43]. The implementation of the intervention was assessed through provider interviews in two of the studies. No other process evaluation methodology was reported.

The rationale for the duration of the intervention was not always apparent in the reporting of the studies. One study provided the following rationale: ‘a 12 week intervention period was perceived by study clinicians and investigators to be typical when treating this population’ [41].

## Discussion

The small number of IHC trials found in this review was disproportionate to the amount of literature identified. This supports the findings of Coulter et al. [20] which concluded that research is still focused on conceptualizing and practicing IHC not on efficacy [9]. The paucity of trials also likely reflects the difficulty in undertaking research on complex interventions such as IHC. However, there is a need for research that evaluates this integration, reflects clinical practice and provides an understanding of efficacy [4, 47]. Reviews or summaries of this evolving field are sparse and few have focused specifically on clinical trials [19, 48]. It is perhaps timely that we examine the reporting, structure and outcomes of recent trials within the context of emerging frameworks for researching complex interventions so as to guide future work in this field.

The theoretical rationale reported for undertaking a clinical trial of an IHC intervention was manifold. Firstly, in ‘real life’, patients frequently don’t restrict their CM use to one therapy and often seek to use a combination [38]. Patients are usually the point at which integration occurs - ferrying results, treatment plans and x-rays between providers. It is argued that the most appropriate point of integration is the provider through IHC [49]. Secondly, there has been much discussion about conceptual IHC models but little testing to examine how IHC may be implemented, at what cost and effectiveness [42]. Thirdly, the combined effect or synergy of multiple interventions is hypothesised as likely to be greater than that which can be achieved by a single modality, particularly for chronic conditions [41]. Provision of an individualised multidisciplinary intervention provides patients with greater choice, and facilitates patient participation in the decision-which is associated with better health outcomes and satisfaction [50, 51].

Not all health conditions or diseases are suited to an IHC intervention. In the trials examined in this paper, lower back pain was the condition selected for four or five trials. It is likely that this was selected for a number of reasons. Lower back pain is a prevalent condition which has a high community and economic cost [52]. Musculoskeletal conditions are one of the most common conditions for which people consult a CM practitioner utilizing both conventional and CM treatments alongside one another [53, 54]. There was a clear intent within the studies to include modalities for which a positive evidence base was available. A recent systematic review and meta-analysis of the efficacy, cost-effectiveness and safety of selected CMs for neck and LBP found that CM treatments such as acupuncture, massage, spinal manipulation and mobilization, were more effective at reducing pain in the short term than no treatment, placebo, physical therapy or usual care [52]. Trials typically examined single CM modalities in isolation, and multidisciplinary approaches rarely included CM therapies. Other types of conditions where IHC interventions might be useful are likely to be those where CM use is high, such as the for the treatment of health problems where there is unmet need and conventional care has not been able to help; that have an unpredictable course and prognosis; and that are associated with substantial pain, discomfort, or side effects from prescription drug medicine [4, 55]. In a recent paper, IHC leaders nominated headache, back pain, arthritis and diseases across the metabolic spectrum as areas where the body of CM evidence was strong and suited an IHC trial [56]. In another study of general practice, conditions such as musculoskeletal problems, depression, eczema, chronic pain, and irritable bowel syndrome were suggested [57].

To understand the IHC interventions – what works and what doesn’t – we need as much transparency as possible. In the studies we reviewed development plans or protocols documented a consistent structure, process and functional intent around the variable components of IHC intervention. The completed trials in the review provided several papers reporting the results of various aspects of the intervention. In the majority of the studies this was sufficient detailed to enable the replication of elements of the design, although a clear parallel evaluation of the process would be of benefit. Only one study in our review included a nested ‘process evaluation’[37]. Process evaluations within trials explore the implementation, receipt, and setting of an intervention and help in the interpretation of the outcome [58]. A process evaluation embedded within future trials may assist in documenting the time taken to construct a patient profile; the skills needed to collect the information for the patient profile; the time needed to devise a treatment plan, present the plan and reach consensus; help to distinguish between essential and non-essential components of the intervention; investigate contextual factors that affect an intervention; patient responsiveness; practitioner delivery; and monitor dose [23, 59, 60].

Equally useful to understanding the IHC interventions is to know what ‘guides’ the treatment planning and management process beyond the integrative care and management. Table 4 includes a set of ‘guiding principles’ articulated by Maiers et al.[61]. These principles serve to clearly delineate the intent or function of the intervention, and the approach the team should aim to take. Of these five principles, all studies were guided by Principles 4, 5 and 6. It was less clear the extent to which studies were guided by the other three principles.

**Table 4:** Guiding principles for an IHC intervention^1^

Minimal intervention approach to treatment to prevent fear & castrophizing
Goal of treatment to decrease the patients’ dependency on the health care system
Limits on treatment should not be arbitrarily applied to care
An evidence informed practice model based on patient presentation, clinical experience and research evidence
Each individual is unique and treatment should be modified accordingly
Integrative multidisciplinary approach to management

Table modified from Maiers [61]

The basic approach to the provision of care in each of the studies was guided by Principle 4: using evidence informed interventions and translate existing complementary and integrative therapies into clinical practice bringing together practitioner expertise, patient presentation and preference to form the treatment plan [62]. In some studies, the organisational process of constructing a treatment plan was documented and this is useful for future replication. Some of the trials then documented the frequency and types of treatments patients received although the details and rationale of the actual treatments are not provided. Journal article length makes this level of reporting unfeasible. Understanding frequency and intensity of the individualised treatments may be graphically depicted in ways suggested by Perera [63]. This may provide an indication of ‘dose’ per individual. The use of an inadequate dose may be safe and less resource intensive but ineffective.

IHC practitioners in all studies were provided with education and training of variable intent and intensity. The provision of education and training prior to study commencement demonstrated an understanding of the complexity of bringing different professional health disciplines together [64]. Opportunities for dialogue between different practitioners and group development build mutual understanding which is considered important for success of IHC [65, 66]. Future studies should consider the informal and formal means for collaboration and team building not only at study commencement but throughout the study. It is likely established IHC teams would operate in a more timely and cost effective fashion. Increased team cohesiveness may lead to improved safety, sharing resources, less opportunity for negative treatment interactions, reduced treatment cycles, reduced primary care visits and cost effectiveness [42].

The studies in our review used disease specific outcome measures combined with a quality of life measure and/or a patient reported measure such as MYMOP. While disease specific measures are important, so too are outcome measures on IHC interventions that cover physical, spiritual, psychological and social domains which go beyond measuring disease specific biomedical outcomes [67, 68]. Combinations of qualitative and quantitative measures are best placed to provide comprehensive outcome data. Philosophically, IHC and CM practices are not typically based on a mechanistic cause-effect relationship with a specific intervention for a specific symptom. Rather the approach is to bring the whole person back into ‘balance’ [69]. One study in our review reported qualitative outcomes relating to changes in self-concept and empowerment, and benefits arising from treatment that was ‘whole-person’ focused [46]. This supports other studies that indicate that IHC is associated with improved health related quality of life in diverse populations with substantial co-morbidity [70]. Including measures that capture these outcomes is particularly relevant if the cost effectiveness of IHC through its capacity to reduce co-morbidities alongside disease specific symptoms and improve total well-being is to be captured [56, 60, 69, 71].

Measuring outcomes and designing IHC interventions is further complicated by understanding that causality lies for the effectiveness of an IHC intervention lies not just with the treatment component but by enhancing the healing capacity of the patient (salutogenesis) through the social context and healing environment [72]. The patient focused, IHC team based approach is thought to enhance this process as team members contribute unique perspectives, skills and experience to patient care [73, 74]. Bell argues that the whole therapeutic strategy of IHC needs to be evaluated: including the patient-provider relationship, multiple conventional and CAM treatments, and the philosophical context of care as the intervention [69]. Only one of the studies in our review included measures to attempt to evaluate this process [75]. Future clinical trials of IHC interventions should include qualitative elements that seek to understand the ways in which this process may be fostered to maximise the specific and nonspecific healing effect of an IHC intervention.

The strengths of an IHC intervention to provide good external validity need to be considered against the inherent limitations of undertaking the evaluation of a complex intervention using an experimental design (see Table 5). Defining and articulating the “black box” of an IHC intervention is important for internal validity, generalizability and replicability [59]. The difficulty in doing so within this type of trial design is one of the key limitations of a complex intervention. The individualised nature of IHC makes it difficult to know which component of the intervention is exerting the main effect - the combination of the therapies, the extra attention, the patient-practitioner relationship or something else not considered.

**Table 5:** Strengths and weakness of IHC intervention studies

Strengths	Challenges/Limitations
Individualised, tailored	Active components are obscured
Aims to heal the whole person	Difficult to replicate
Suits chronic conditions	Poor internal validity
Good external validity	Not readily transferrable to other sites as dependent on availability of modalities, certification of providers, cost
Potential to reduce health costs	May require a long trial period with follow up to establish efficacy and cost effectiveness
Non-specific benefits due to increased attention, health literacy and education	Non-specific benefits may be practitioner dependent.

For the purposes of research, IHC is a therapeutic strategy not a single drug intervention. Team-based, patient-centred, integrative approaches to care present a challenge to designing rigorous studies, given IHC is typically used to provide many simultaneous treatments for multiple health concerns [68]. Many efforts have been made to propose frameworks for researching complex healthcare such as IHC [28, 59, 60, 69] calling on program theory [73], whole system theory [69, 72], utilising the Medical Research Council (MRC) framework or employing implementation, process or fidelity evaluation [59]. Research methodology for evaluating IHC probably best involves a combination of understanding the philosophical underpinnings of IHC through whole system theory and examining it within the MRC framework. Whole systems can be defined as “approaches to healthcare in which practitioners apply bodies of knowledge and associated practices in order to maximize the patients’ capacity to achieve mental and physical balance and restore their own health, using individualised, non-reductionist approaches to diagnosis and treatment”[76]. In the case of IHC, it is the individualised integrative therapeutic strategy that is the whole system intervention.

The MRC framework follows a typical drug development pathway but provides guidance for identifying confounders, modelling to predict how components may interact and identifying the constant and variable components of the intervention. Within the MRC framework a nested “process evaluation” within the study would provide insight on the constant and variable components of the intervention. A process evaluation would investigate contextual factors such as setting, team composition and facilitation, and examine patient-provider expectations and relationships [24]. A process evaluation may follow some of the dimensions identified in program implementation theory: fidelity, quality of delivery, participant responsiveness, and program adaptation [77] Each of these dimensions has been demonstrated to influence outcomes. Strategies may include interviews, focus groups, and observations alongside document reviews of clinical files and correspondence. An additional complexity is that, not unlike conventional interventions for chronic and complex conditions, research needs to be conducted over the longer term to truly capture outcomes. Jonas et al. [72] document an evaluation model for integrative care specifically for cancer but equally applicable to understanding and evaluating IHC in primary care . The model is designed to collect data on wellbeing, behavior, clinical outcomes, bio-measures, costs and the course of treatment and compare IHC with standard healthcare practices.

A further challenge in IHC research is the preference of researchers and funders typically prefer investigations that are linear, showing a clear cause and effect, with few confounders and cost effectiveness can clearly be determined [4] . In the US Institute of Medicine examination of contemporary approaches to CM research acknowledge the limitations of researchers trained in Western cultures, where complex “systems” with multiple levels of relationships and multiple factors which are interactive and iterative and do not fit into this preferred type of research [4]. The typical efficacy focused RCT prescribes to the ‘average’ patient and is ‘fundamentally’ at odds with CM orientation to the ‘individual’ patient . The number of patients using CM continues to grow, as does the number of patients that desire a general practitioner who communicates about, and refers to, CM practitioners. Patients are seeking care that is tailored to their individual needs and where CM and conventional medicine collaborate [78–81] with availability of these treatment options in one location being cited as desirable [78]. These considerations aside, patients are already integrating conventional and CM therapies on their own due to a desire to access the best that both healthcare paradigms have to offer [5]. Investigations on the efficacy, safety and cost effectiveness of an IHC model of care are warranted to guide health policy makers and consumers in decision-making and there are sufficient research and statistical methods available that enable such investigation.

Proposed RCT designs for complex interventions include pragmatic trials, factorial design, preference trials and randomised consent designs, N-of-1 designs [28]. These trial designs may be used to address the preferences of patients, which are often strong in CM users, for an integrative approach [82]. In considering comparators and research design, there is a broad consensus that the evaluation of IHC and CM be conducted where possible within a comparative effectiveness framework [28, 83, 84]. The Institute of Medicine defines CER as “the generation and synthesis of evidence that compares the benefits and harms of alternative methods to prevent, diagnose, treat, and monitor a clinical condition or to improve the delivery of care [85]. In selecting comparators for an experimental design, IHC lends itself with some ease to comparative effectiveness studies.

The cost effectiveness data collected in the studies in our review was comprehensive in only one study. Cost effectiveness analysis for IHC interventions should include sick leave, medication use, return-to-work and other cost-related data alongside cost of treatment course, and compliance. Sundberg et al. [42] recommend future studies include collection of cost through using cost diaries, measuring health care visits, sick leave.

From the reviewed studies, it is difficult to determine the type and level of resources required to conduct IHC patient assessment and treatment planning. Patient assessment and enrolment in the trials were typically undertaken by one or two therapists this may increase cost. Likewise means for collaboration such as meetings are costly. Any additional duties typically come at a direct financial cost to fee-for-service practitioners or need to be compensated for within the IHC model. Conversely it is thought that within the integrative whole person approach there is considerable potential for cost-effectiveness [56]. Some preliminary data shows that various CM interventions may be cost effective [86]. Consumers and policy makers need to know if IHC models are cost effective for effective decision-making.

This review provides an opportunity to consider methodological challenges that arise in undertaking a trial of a multidisciplinary, complex intervention. To enable an understanding of how an individualised intervention is developed the structure and the process utilised needs to be transparent. With single interventions, internal validity is maximised by having a standard dose delivered in the same way at each trial site. Complex interventions can retain some internal validity by standardising the process, guiding principles, and structure of the intervention while allowing the ‘form’ to be adapted [87].

A limitation of our review is that we may have omitted some studies. Our review is also limited by the inclusion criteria we selected. Our review supports the findings of Coulter et al., that the lack of a clear definition and taxonomy for integrative health care makes reviewing this field challenging [19].

## Conclusions

The trials in our review provide a small yet critical base from which to draw upon, refine and develop larger studies. We recommend pragmatic controlled trials that document the structure of the IHC environment, the process of care and measure outcomes beyond those reported in mechanistic efficacy based trials similar to the efforts being undertaken in the Integrative Medicine PrimAry Care Trial (IMPACT) [59]. Complex interventions have inherent limitations not the least of being low internal validity. To strengthen the quality of the trials, trials should have built in process evaluations and clear guiding principles that provide structure beyond the individualized treatment components. Such trials will provide evidence of efficacy, safety and identify challenges and opportunities of this healthcare strategy. The trials will complement the large scale observational studies of IHC interventions such as that conducted on Chronic Pain by the Bravewell Integrative Medicine Research Network (BRAVENET) collaborators [88]. An evidence base on IHC is needed to inform patients and healthcare providers and policy makers. Future research in the IHC field would do well to build on the recommendations from the existing body of experimental design. In chronic illnesses where there is enormous burden there is considerable merit in investigating an IHC approach to see if it is able to reduce the health costs.
